# Hyperspectral data of understory elements in boreal forests: In situ and laboratory measurements

**DOI:** 10.1016/j.dib.2024.111068

**Published:** 2024-10-24

**Authors:** Audrey Mercier, Susanna Karlqvist, Aarne Hovi, Miina Rautiainen

**Affiliations:** School of Engineering, Aalto University, Espoo, Finland

**Keywords:** Reflectance and transmittance factors, Ferns, Wood sorrel, Maianthemum bifolium, Decaying wood, European fly honeysuckle, Alder buckthorn, Common hazel

## Abstract

Enhancing our understanding of the spectral properties of forest elements is essential for interpreting airborne and satellite-borne remote sensing data. This article presents two datasets on the spectral properties of understory elements in boreal forests collected with close-range hyperspectral measurements. We conducted two field campaigns in June and July 2023 in Finland to acquire spectral measurements at wavelengths from 350 to 2500 nm using an ASD FieldSpec 4 spectrometer for forest understory elements. We measured ferns, decaying wood, common wood sorrel and May lily in situ. In a laboratory, we measured leaves from European fly honeysuckle, alder buckthorn and common hazel. These data support the analysis of vegetation characteristics, training of classification algorithms and improvement of forest radiative transfer models, and could be used to evaluate the potential of hyperspectral data to discriminate the understory elements of boreal forest.

Specifications Table

**Table:** 

Subject	Forestry
Specific subject area	In situ and laboratory measurements of spectral properties of understory elements of boreal forest.
Type of data	Table (.csv format) Supporting materials (.pdf format) Raw
Data collection	The data were collected in Finland in June and July 2023 using a spectroradiometer (ASD FieldSpec 4 Standard-Res). The reflectance spectra of forest floor elements (ferns, *Oxalis acetosella, Maianthemum bifolium*, and decaying wood) were measured in situ. Leaves were collected from three understory bush species (*Lonicera xylosteum* L., *Frangula alnus*, Mill., *Corylus avellana* L.), and their reflectance and transmittance spectra were measured in a laboratory. Each measurement was associated with spatial coordinates and metadata describing the sample (i.e., the individual element of the understory measured using the spectroradiometer) and its surrounding environment.
Data source location	Three locations around the Hyytiälä forest station, Hyytiäläntie 124, 35,500 Korkeakoski, Finland (61° 51′N, 24° 18′E) Viikki arboretum, 00790 Helsinki, Finland (60° 13′N, 25° 00′E)
Data accessibility	Repository name: Mendeley **Reflectance spectra of boreal forest floor elements: ferns, herbaceous plants and decaying wood** [3]: We will call this dataset the “forest floor elements dataset” for the sake of brevity. Data identification number: https://doi.org/10.17632/dddb2prk4p.1 Direct URL to data: https://data.mendeley.com/datasets/dddb2prk4p/1 **Leaf spectra of alder buckthorn, common hazel and European fly honeysuckle** [4]: We will call this dataset the “bush leaves dataset” for the sake of brevity. Data identification number for bushes: https://doi.org/10.17632/3bkrwhk4p3.1 Direct URL to data: https://data.mendeley.com/datasets/3bkrwhk4p3/1
Related research article	none

## 1 Value of the Data

• Field and laboratory measurements of spectral properties of various forest elements are needed to form the scientific basis for remote sensing-based monitoring of biodiversity. However, progress in hyperspectral remote sensing has been limited by an incomplete understanding of spectral variations in forest elements. Previously, more focus has been placed on measuring the spectral properties of economically profitable species (e.g., trees, crops). These new datasets provide spectra of forest elements that have been understudied until now.
• These data can be used as input in forest radiative transfer models and to train machine learning based methods for the interpretation of multi- or hyperspectral remote sensing data.
• These spectral libraries can be used to study the spectral diversity of forest elements and can be integrated into digital twins of forests.

## 2 Background

In boreal forest ecosystems, forest floor and understory components are an integral part of biodiversity and play a crucial role in various ecological processes. Decaying wood is particularly important for biodiversity, for example, serving as shelter and food resources for multiple animal species. Ferns, herbs and bushes, on the other hand, can serve as ecological indicators to describe the habitat quality. Remote sensing is a promising tool for characterizing and monitoring forest ecosystems at spatial scales ranging from local to global. Spectral libraries play a crucial role in the remote sensing process by describing the spectral signature of various elements that are crucial for characterizing forest biodiversity. While existing spectral libraries in boreal forests focus mainly on forest canopy elements such as tree leaves or needles, they are limited concerning the understory elements. Previous studies using hyperspectral data have shown that forest floor partly contributes to the signal recorded at the pixel level [1,5]. Therefore, it is necessary to measure and publish spectral libraries on understory elements and include a more diverse range of sample types (i.e., species, habitats).

## 3 Data Description

Two datasets are associated with this article: one for forest floor elements measured in situ and the other for bush leaves measured in a laboratory.

**Fig. 1:**
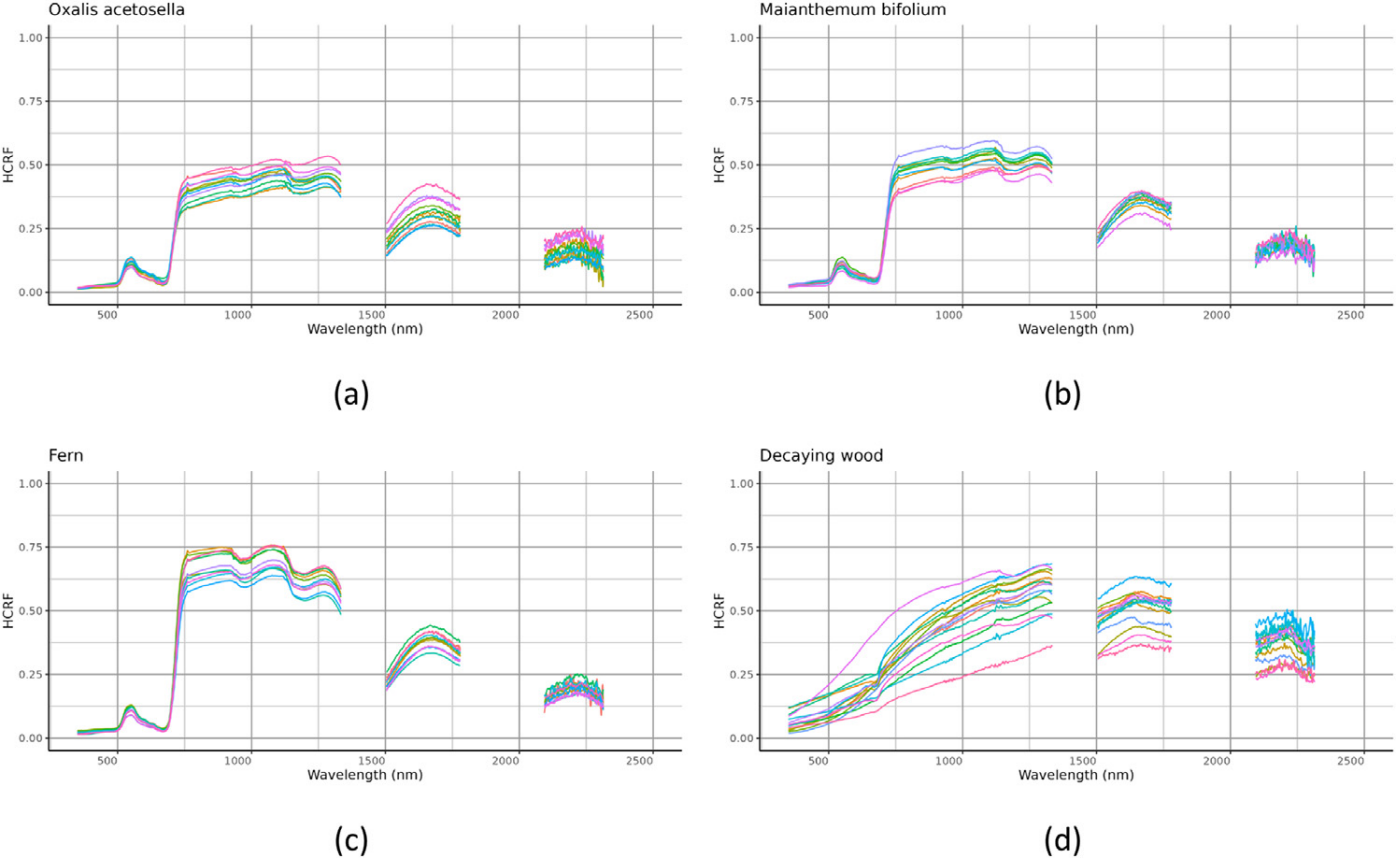
Hemispherical-conical reflectance factors (HCRF) measured for (a) common wood sorrel (*Oxalis acetosella)*, (b) May lily (*Maianthemum bifolium)*, (c) fern and (d) decaying wood. HCRFs from 1336 to 1504 nm, 1781 to 2094 nm and 2316 to 2500 nm were excluded due to atmospheric attenuation causing noise.
(3) a table named “description.csv” containing metadata per sample (see Table 1 for details).

The “forest floor elements dataset” includes:

(1) a document named “README.pdf” detailing the data collection, the measurement process and the content of the data files.
(2) a table named “spectral measurements.csv” containing hemispherical-conical reflectance factors (HCRF) with one sample (i.e., an individual element of the understory measured using the spectroradiometer) per row. The first column is the unique ID of the sample and the 2150 other columns named “wl [wavelength in nanometer]” correspond to the HCRF measured at the spectral bands from 350 to 2500 nm. Fig. 1 shows the HCRF spectra of the samples in the dataset, the R code used to produce this figure is included as a supplementary file.

**Table 1:** Column names and corresponding metadata for the “description.csv” file included in the forest floor elements dataset.

Column name	Description
sample ID	Sample unique identifier
X_coord	Longitude value of sample position in WGS84 coordinate system (EPSG:4326)
Y_coord	Latitude value of sample position in WGS84 coordinate system
site	Location identifier (1, 2, 3).
target_type	Type of forest element: • dw: decaying wood sample • fe: fern • ma: *Maianthemum bifolium* (L.) F.W. Schmidt • ox: *Oxalis acetosella* L.
date	Date of the measurement (YYYYMMDD)
forestcover	Visual estimate of overstory canopy cover above the sample (0 - 100 %)
dominanttreespecies	Dominant tree species in the immediate environment surrounding the sample
(4) a document named “sample_photos.pdf” showing photographs of the samples and their surrounding environment (Fig. 2).

**Fig. 2:**
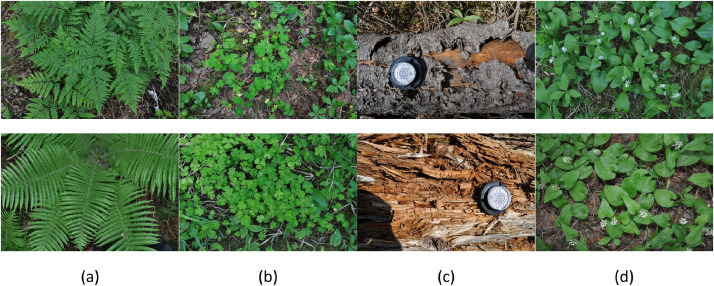
Examples of photographs of samples measured for (a) fern, (b) common wood sorrel (*Oxalis acetosella*), (c) decaying wood, (d) May Lily (*Maianthemum bifolium*).

**Fig. 3:**
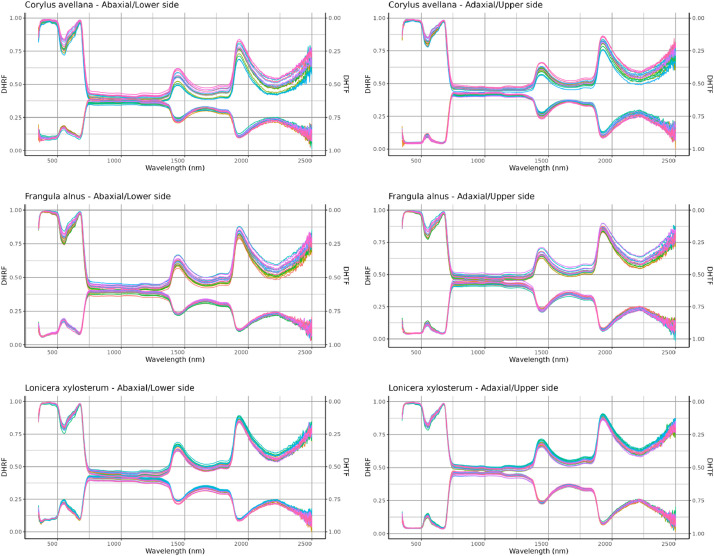
Directional-hemispherical reflectance (DHRF) (lower signatures) and transmittance factors (DHTF) (upper signatures) measured on abaxial (left column) and adaxial (right column) sides of leaves collected from common hazel (*Corylus avellana*), alder buckthorn (*Frangula alnus*) and European fly honeysuckle (*Lonicera xylosteum*).
(3) a table “description.csv” containing the metadata for each sampled leaf (see Table 2 for details).

The “bush leaves dataset” includes:

(1) a document named “README.pdf” detailing the data collection, the measurement process and the content of the data files.
(2) four tables containing the spectral measurements. The “spectra R adaxial.csv” and “spectra R abaxial.csv” contains directional-hemispherical reflectance factors (DHRF) of the adaxial and abaxial leaf sides, respectively. The “spectra T adaxial.csv” and “spectra T abaxial.csv” contains directional-hemispherical transmittance factors (DHTF) of the adaxial and abaxial leaf sides, respectively. The first column of those files is the unique ID of the measured leaf and the 2150 other columns named “wl [wavelength in nanometer]” correspond to the DHRF/DHTF measured at the spectral bands from 350 to 2500 nm. Fig. 3 shows the DHRF and DHTF spectra of the samples in the dataset, the R code used to produce this figure is included as a supplementary file.

**Table 2:** Column names and corresponding metadata for the “description.csv” file included in the bush leaves dataset.

Column name	Description
sample_ID	Leaf unique identifier
bush_ID	Bush unique identifier
X_coord	Longitude value of the sampled bush position in WGS84 coordinate system (EPSG:4326)
Y_coord	Latitude value of sample position in WGS84 coordinate system
species	Bush species: • C: Corylus avellana L. • F: Frangula alnus Mill. • L: Lonicera xylosteum L.
date	Date of the measurement (YYYYMMDD)
(4) a document named “sample_photos.pdf” showing photographs of the sampled leaves, branches and bushes, as well as their surrounding environment (Fig. 4).

**Fig. 4:**
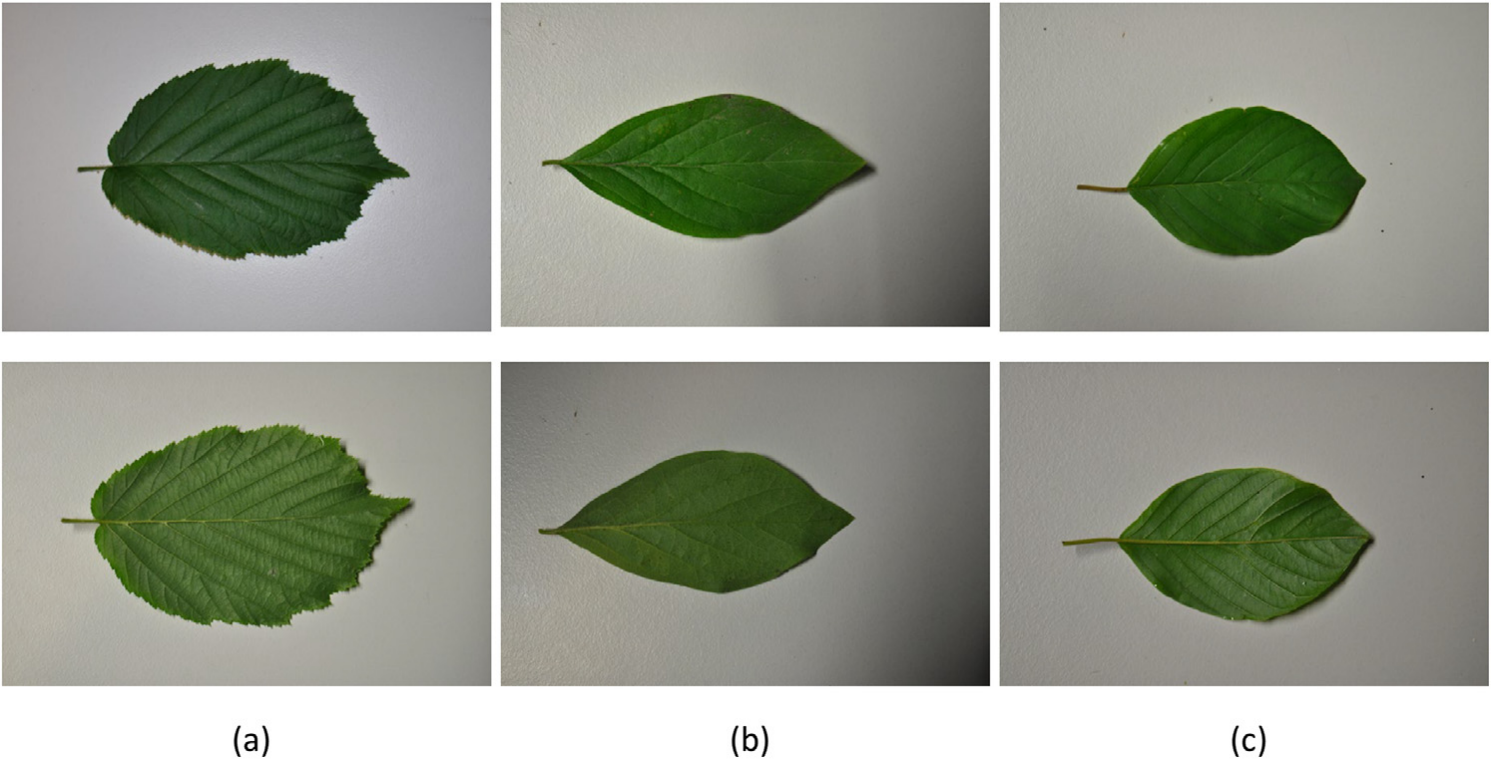
Examples of photographs of sampled leaves collected from (a) common hazel (*Corylus avellana*), (b) alder buckthorn (*Frangula alnus*) and (c) European fly honeysuckle (*Lonicera xylosteum*). Upper and lower rows correspond to adaxial (upper) and abaxial (lower) sides of the leaf, respectively.

## 4 Experimental Design, Materials and Methods

Laboratory measurements of leaf reflectance and transmittance spectra were conducted from 5 to 10 July 2023 for samples of European fly honeysuckle (*Lonicera xylosteum* L.), alder buckthorn (*Frangula alnus* Mill.) and common hazel (*Corylus avellana* L.) collected from the Viikki arboretum, in Helsinki, Finland (60° 13′N, 25° 00′E). Leaves were obtained from three bushes per species, six branches per bush and one leaf per branch. After cutting, the branches were placed in tubes filled with water and stored in cool conditions until the leaf spectra were measured (within 7 h of sample collection). Each sampled leaf was detached from the branch just prior to measurement, systematically selected and required to be fully developed and healthy. Directional-hemispherical reflectance and transmittance factors (DHRF and DHTF, respectively) were measured in a darkroom using an Analytical Spectral Devices (ASD) RTS-3ZC integrating sphere attached to a spectrometer (ASD FieldSpec 4 Standard-Res, serial number 18,456). DHRF and DHTF were measured at wavelengths from 350 to 2500 nm. The spectral resolution was 3 nm in the visible to near-infrared wavelengths (≤ 1000 nm) and 10 nm in the shortwave-infrared wavelengths (>1000 nm). The measurements were interpolated at 1 nm intervals by the instrument. DHRF and DHTF were measured on the adaxial (upper) and abaxial (lower) sides of the leaf and calculated using the following equations:

(1) $$DHRF\left(\lambda \right)=\frac{\left(DN_{s,R}\left(\lambda \right)-DN_{dc}\left(\lambda \right)\right)-\left(DN_{str,R}\left(\lambda \right)-DN_{dc}\left(\lambda \right)\right)}{\left(DN_{ref,R}\left(\lambda \right)-DN_{dc}\left(\lambda \right)\right)-\left(DN_{str,R}\left(\lambda \right)-DN_{dc}\left(\lambda \right)\right)}*R_{ref}\left(\lambda \right)$$

(2) $$DHTF\left(\lambda \right)=\frac{DN_{s,T}\left(\lambda \right)-DN_{dc}\left(\lambda \right)}{DN_{ref,T}\left(\lambda \right)-DN_{dc}\left(\lambda \right)}*R_{ref}\left(\lambda \right)$$

where *DN_s,r_* and *DN_ref,R_* are the wavelength dependent (*λ*) spectrometer readings from the DHRF measurement of the sample and white reference measurement for DHRF, respectively. *DN_s,T_* and *DN_ref,T_* are the spectrometer readings from the DHTF measurement of the sample and white reference measurement for DHTF. *DN_d,c_* is the dark current and *DN_str,R_* the stray light reading for DHRF measurements. *R_ref_* is the DHRF of the white reference panel. A bias correction was applied to DHTF measurements by multiplying the resulted DHTF values by 0.945 [2].

In situ spectral measurements were collected, from 19 to 29 June 2023, for 11 fern plots, 11 common wood sorrel (*Oxalis acetosella*) plots, 10 May lily (*Maianthemum bifolium*) plots and 15 decaying wood samples close to the Hyytiälä forest station in Finland (61° 51′N, 24° 18′E). Hemispherical-conical reflectance factors (HCRF) were measured using a spectrometer (ASD FieldSpec 4 Standard-Res, serial number 18,456) and a white reference panel (a calibrated 25 × 25 cm Spectralon® diffuse reflectance target with 99 % nominal reflectance) at wavelengths from 350 to 2500 nm at the same intervals as laboratory measurements. Each understory element was measured from at least three samples per location under diffuse illumination during daytime (6 a.m to 8 p.m.). Samples were cleaned prior to measurements by removing the most dissimilar elements. Next, three measurements per sample were performed: a white reference, a sample and a dark current measurement. The receiver was a fiber-optic cable with a conical field-of-view with an opening angle of 25°. The distance between the fiber-optic cable and the sample was 16, 22, 45 cm for decaying wood samples, ferns and herbs (common wood sorrel and May lily), corresponding to circular fields of view with diameters of 7, 10, 20 cm, respectively. The HCRF was computed using the following equation:

(3) $$HCRF\left(\lambda \right)=\frac{DN_{sample}\left(\lambda \right)-DN_{dc}\left(\lambda \right)}{DN_{wr}\left(\lambda \right)-DN_{dc}\left(\lambda \right)}*R_{ref}\left(\lambda \right)$$

where DNs are the wavelength dependent (*λ*) signal values from the sample (sample), the white reference panel (wr) and the dark current (dc) measurements, respectively. *R_ref_* is the reflectance factor of the white panel used as a correction term to account for the non-ideality of the panel's reflectance properties.

The spatial coordinates of the floor elements and bushes were recorded in WGS84 coordinate system (EPSG:4326) using a handheld navigator (Garmin GPSMAP® 62stc) with an accuracy of 3 to 5 m.

## Limitations

None.

## Ethics Statement

The current work does not involve human subjects, animal experiments, or any data collected from social media platforms.

## CRediT authorship contribution statement

**Audrey Mercier:** Methodology, Software, Investigation, Data curation, Writing – original draft, Visualization. **Susanna Karlqvist:** Investigation, Data curation, Writing – review & editing. **Aarne Hovi:** Methodology, Software, Writing – review & editing. **Miina Rautiainen:** Conceptualization, Methodology, Writing – review & editing, Supervision, Project administration, Funding acquisition.

## Data Availability

Mendeley DataLeaf spectra of alder buckthorn, common hazel and European fly honeysuckle (Original data)
Mendeley DataReflectance spectra of boreal forest floor elements: ferns, herbaceous plants and decaying wood (Original data)

## Appendix Supplementary materials

Image, application 1

## References

[1] Eriksson H.M., Eklundh L., Kuusk A., Nilson T. (2006). Impact of understory vegetation on forest canopy reflectance and remotely sensed LAI estimates. Remote Sens. Environ..

[2] Hovi A., Mõttus M., Juola J., Manoocheri F., Ikonen E., Rautiainen M. (2020). Evaluating the performance of a double integrating sphere in measurement of reflectance, transmittance, and albedo of coniferous needles. Silva Fennica.

[3] Mercier A., Karlqvist S., Hovi A., Rautiainen M. (2023). Reflectance spectra of boreal forest floor elements: ferns, herbaceous plants and decaying wood. Mendeley Data.

[4] Mercier A., Karlqvist S., Hovi A., Rautiainen M. (2023). Leaf spectra of alder buckthorn, common hazel and European fly honeysuckle. Mendeley Data.

[5] Rautiainen M., Lukeš P. (2015). Spectral contribution of understory to forest reflectance in a boreal site: an analysis of EO-1 Hyperion data. Remote Sens Environ.

