# Supplementary material for: Hyperspectral data of understory elements in boreal forests: In situ and laboratory measurements
Source: Data Brief. 2024 Oct 24;57:111068. doi: 10.1016/j.dib.2024.111068 (PMC11570963; doi:10.1016/j.dib.2024.111068)

R script to produce Figure 1

Hemispherical-conical reflectance factors (HCRF) measured for common wood sorrel (*Oxalis acetosella*), May lily (*Maianthemum bifolium*), fern and decaying wood. HCRFs from 1336 to 1504 nm, 1781 to 2094 nm and 2316 to 2500 nm were excluded due to atmospheric attenuation causing noise.

rm(list=ls())
gc()

library("ggplot2")
library("tidyverse")

# Read spectral measurements
spt <- "YOUR_PATH//spectral_measurements.csv" |>
 read.csv(skip=2)

# Exclude noisy regions
spt[,(c(1336:1504,1781:2094,2316:2500)+1-350)]=NA

# Format table
spt=spt |>
 pivot_longer(-1) |>
 mutate(band=as.numeric(gsub('wl','',name)),
 target=substr(sample_ID,1,2))

# Define a ggplot object template in a new function
ggplot_for_loop <- function(targetname,title) {
 ggplot(data=subset(spt,target==targetname),aes(x=band,y=value,group=sample_ID,color=sample_ID))+
 geom_line()+
 ylim(0,1)+
 ggtitle(title)+
 xlab("Wavelength (nm)")+
 ylab("HCRF")+
 theme(
 plot.background = element_rect(fill = "white"),
 panel.background = element_rect(fill = "white"),
 panel.grid.major.y = element_line(color = "grey60",linewidth=0.5),
 panel.grid.major.x = element_line(color = "grey60",linewidth=0.5),
 panel.grid.minor.x = element_line(color = "grey80",linewidth=0.5),
 panel.grid.minor.y = element_line(color = "grey80",linewidth=0.5),
 axis.line = element_line(colour = "black"),
 legend.position="none",
 axis.text.x = element_text(angle = 0, vjust = 1, hjust=1))
}

# Display ggplot by type of forest element
targetname_lst=c("fe","dw","ma","ox")
title_lst=c("Fern","Decaying wood","Maianthemum bifolium","Oxalis acetosella")
for (i in 1:4){
 print(ggplot_for_loop(targetname=targetname_lst[i],title=title_lst[i]))
}


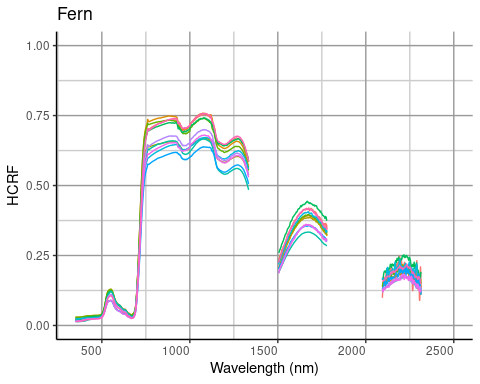


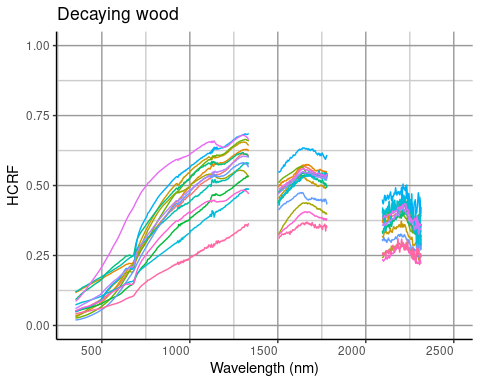


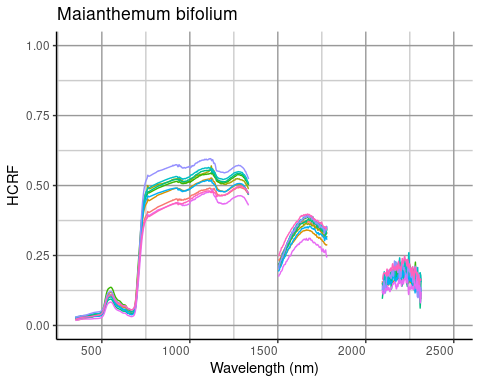


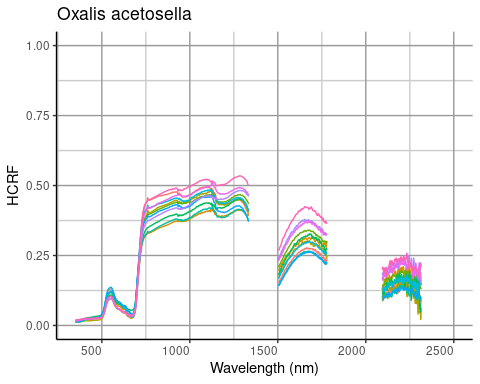


R script to produce Figure 3

Directional-hemispherical reflectance (DHRF) (lower signatures) and transmittance factors (DHTF) (upper signatures) measured on abaxial (left column) and adaxial (right column) sides of leaves collected from common hazel (*Corylus avellan*a), alder buckthorn (*Frangula alnus*) and European fly honeysuckle (*Lonicera xylosteum*)

rm(list=ls())

library("ggplot2")
library("tidyverse")

# Read spectral measurements
path <- 'YOUR_PATH'
fls <- dir(path=path,full.names = T, pattern='spectra')

# Read and format tables containing DHRF
R_abax <- str_subset(fls, 'R_abax') |>
 read.csv(skip=2) |>
 pivot_longer(-sample_ID) |>
 mutate(band=as.numeric(gsub('wl','',name)),type="Reflectance")
R_adax <- str_subset(fls, 'R_adax') |>
 read.csv(skip=2) |>
 pivot_longer(-sample_ID) |>
 mutate(band=as.numeric(gsub('wl','',name)),type="Reflectance")
# Read and format tables containing DHTF
T_abax <- str_subset(fls, 'T_abax') |>
 read.csv(skip=2) |>
 pivot_longer(-sample_ID) |>
 mutate(band=as.numeric(gsub('wl','',name)),value=1-value,type="Transmittance")
T_adax <- str_subset(fls, 'T_adax') |>
 read.csv(skip=2) |>
 pivot_longer(-sample_ID) |>
 mutate(band=as.numeric(gsub('wl','',name)),value=1-value,type="Transmittance")

# Combine tables containing values for adaxial and abaxial leaf sides
R_T_adax=rbind(R_adax,T_adax)
R_T_abax=rbind(R_abax,T_abax)

# Define a ggplot object template in a new function
ggplot_for_loop<-function(df,shrub_type,title){
 ggplot(data=subset(df, grepl(shrub_type,sample_ID)),aes(x=band,y=value,color=sample_ID, shape=type))+
 geom_line()+
 ylim(0,1)+
 ggtitle(title)+
 xlab("Wavelength (nm)")+
 ylab("DHRF")+ scale_y_continuous(sec.axis = sec_axis(~1-., name="DHTF")) +
 scale_y_continuous(sec.axis = sec_axis(~1-., name="DHTF")) +
 theme(
 plot.background = element_rect(fill = "white"),
 panel.background = element_rect(fill = "white"),
 panel.grid.major.y = element_line(color = "grey60",linewidth=0.5),
 panel.grid.major.x = element_line(color = "grey60",linewidth=0.5),
 panel.grid.minor.x = element_line(color = "grey80",linewidth=0.5),
 panel.grid.minor.y = element_line(color = "grey80",linewidth=0.5),
 axis.line = element_line(colour = "black"),
 legend.position="none",
 axis.text.x = element_text(angle = 0, vjust = 1, hjust=1))
 }

# Display ggplot by bush species and adaxial/abaxial leaf side
shrub_type_lst=c("L_","C_","F_")
title_lst=c("Lonicera xylosterum","Corylus avellana","Frangula alnus")
for (i in 1:3){print(ggplot_for_loop(df=R_T_adax,shrub_type=shrub_type_lst[i],title=paste0(title_lst[i]," - Adaxial/Upper side"))) print(ggplot_for_loop(df=R_T_abax,shrub_type=shrub_type_lst[i],title=paste0(title_lst[i]," - Abaxial/Lower side")))
}


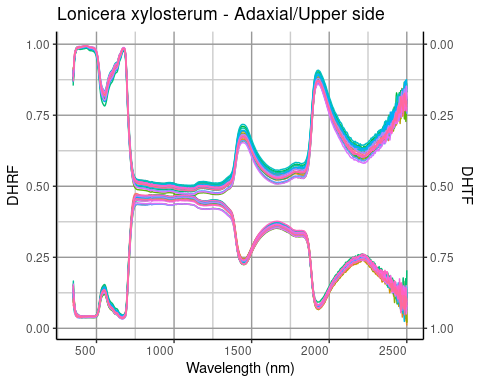


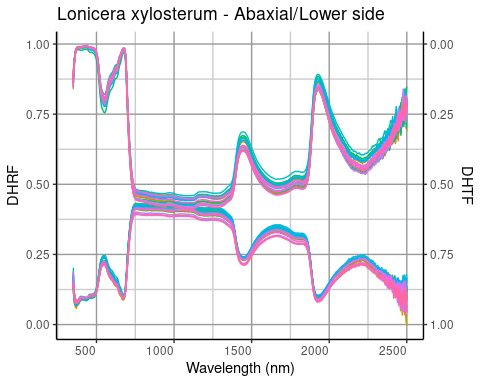


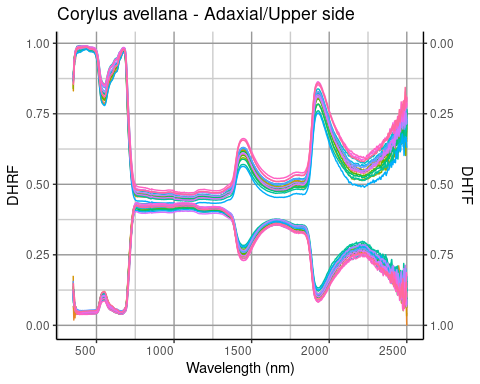


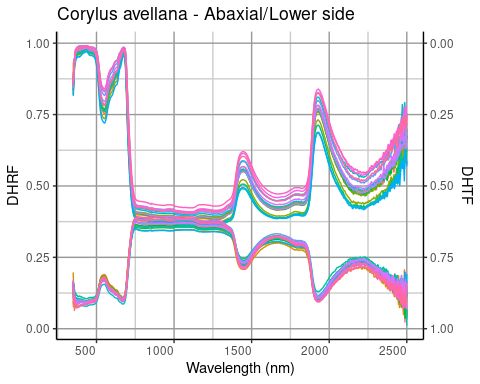


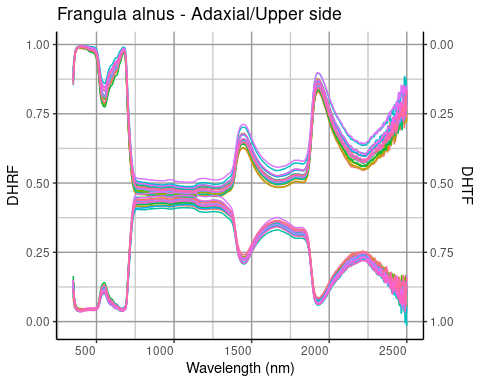

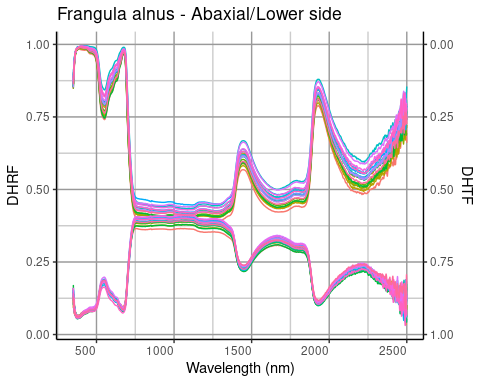

Supplement: Supplementary file 1 [file mmc1.docx]
